# AI Based Clinical Decision-Making Tool for Neurologists in the Emergency Department

**DOI:** 10.3390/jcm14176333

**Published:** 2025-09-08

**Authors:** Alon Gorenshtein, Shiri Fistel, Moran Sorka, Gregory Telman, Raz Winer, Shlomi Peretz, Dvir Aran, Shahar Shelly

**Affiliations:** 1 Department of Neurology, Rambam Health Care Campus, Haifa 3109601, Israel; a_gorenshtein@rambam.health.gov.il (A.G.); s_fistel@rambam.health.gov.il (S.F.); g_telman@rambam.health.gov.il (G.T.); r_winer@rambam.health.gov.il (R.W.); 2 Azrieli Faculty of Medicine, Bar-Ilan University, Safed 1311502, Israel; 3 AI in Neurology Laboratory, Ruth and Bruce Rapaport Faculty of Medicine, Technion Institute of Technology, Haifa 3525408, Israel; m_sorka@rambam.health.gov.il; 4 Department of Neurology, Shamir Medical Center, Tzrifin 72760, Israel; shlomip@shamir.gov.il; 5 Sackler Faculty of Medicine, Tel Aviv University, Tel Aviv 69978, Israel; 6 Faculty of Biology, Technion-Israel Institute of Technology, Haifa 3200003, Israel; dviraran@technion.ac.il; 7 The Taub Faculty of Computer Science, Technion-Israel Institute of Technology, Haifa 3200003, Israel; 8 Department of Neurology, Mayo Clinic, Rochester, MN 55905, USA

**Keywords:** large language models, neurology, predictive models, emergency department, artificial intelligence

## Abstract

**Introduction**: We aimed to prove integration of advanced machine learning methods within a robust ensemble framework can enhance clinical decision-support for neurologists managing patients in the emergency department (ED). **Methods**: We engineered an ensemble framework leveraging the capabilities of the Gemini 1.5-pro-002 large language model (LLM). The model was enhanced using prompt engineering and retrieval-augmented generation (RAG). Predictive modeling achieved by combining eXtreme Gradient Boosting (XGBoost) and logistic regression for optimal accuracy in clinical decision-making. Key clinical outcomes, such as admission and mortality, were assessed. A random subset of 100 cases was reviewed by three senior neurologists to evaluate the alignment of the AI’s predictions with expert clinical judgment. **Results**: We retrospectively analyzed 1368 consecutive ED patients who underwent neurological consultations, assessing their clinical features, diagnostic tests, and admission outcomes. Patients admitted were typically older and had higher mortality rates, shorter intervals to neurological evaluation, and a higher incidence of acute stroke compared to those discharged. For the primary analysis (*n* = 250), the Neuro artificial intelligence (AI) model demonstrated significant performance metrics, achieving an area under the curve (AUC) of 0.88 for general admission predictions in comparison to actual outcomes, an AUC of 0.86 for neurological department admissions, 0.93 for long-term mortality risk, and 1 for 48 h mortality risk. Our Neuro AI model predictions showed a strong correlation with expert consensus (Pearson correlation 0.79, *p* < 0.001), indicating its ability to provide consistent support amid divergent clinical opinions. **Conclusions**: Our Neuro AI model accurately predicted hospital admissions (AUC = 0.88) and neurological department admissions (AUC = 0.86), demonstrating strong alignment with expert clinical judgment.

## 1. Introduction

Annually, approximately 155 million visits are made to the emergency department (ED) in the US [1], accentuating their role as one of the most overburdened components of the healthcare system. ED overcrowding, driven largely by inpatient boarding due to insufficient hospital-wide capacity, significantly contributes to impaired patient flow and resource allocation [2]. Overcrowding and avoidable hospital admissions significantly impact healthcare systems, resulting in increased morbidity, diminished treatment quality, and financial losses [3]. Conversely, premature discharges can lead to return visits from misdiagnosed patients [4], which escalates the risk of medical errors [5]. There are many causes for the above, mainly cognitive factors and mental biases [6,7]. The neurological field faces similarly troubling challenges. The prevalence of neurological disorders is increasing, alongside advancements in biological treatments [8]. Recent studies have even questioned the necessity of neurologists in the ED, further complicating the landscape of neurological care [9,10,11].

Artificial intelligence (AI) is increasingly integrated in conjunction with human systems [12], enhancing efficiency and decision-making across various domains [13,14,15]. In clinical decision-making, integrating an AI copilot at critical junctures can significantly support clinicians by enhancing medical decision-making, interpreting diagnostic results, streamlining ED workflows, facilitating discharge planning, and improving follow-up care. Since human judgment is prone to cognitive biases and limitations, AI tools that augment decision-making hold the potential to improve patient outcomes and reduce errors effectively [16]. Up to now, there have been studies on the use of AI in the ED, but they primarily focused on addressing a single decision and not specifically neurology [17,18,19]. Similarly, within neurology, AI applications have typically concentrated on specific neurological diseases [20], often due to the reliance on deep learning, which requires large amounts of data to achieve adequate performance within a particular problem domain [21]. Consequently, research on AI solutions that address multiple critical decision points in the ED or offer comprehensive support for a wide spectrum of neurological conditions remains limited.

We hypothesize that key decision points in the management of neurological cases in the ED can be effectively optimized through a combination of large language model (LLMs) and traditional machine learning (ML) models. To this end, we propose the Neuro-Copilot, an ensemble framework designed to provide precise, context-aware decision support to ED neurologists and triage teams, aiding in critical tasks such as patient admission or discharge decisions.

## 2. Materials and Methods

### 2.1. Standard Protocol Approvals, Registrations, and Patient Consents

This study was conducted with institutional research board approval (RMB 0026-24).

### 2.2. Cohort Identification

This retrospective study comprised 1368 consecutive cases from the emergency department. Of these, 1118 encounters comprised the training set, and a fixed hold-out test set of 250 “detailed cohort” encounters contained richer annotations used only for evaluation. Clinical information was uniformly extracted using an electronic record retrieval system capable of accessing all clinical and laboratory results. We identified all patients who underwent neurological consultation in the ED from 1 January 2024 to 29 February 2024, with follow-up concluding on 16 August 2024. Inclusion criteria included patients above 18 years old with a medical history. Exclusion criteria included patients below 18 years old, lack of follow-up data until 16 August 2024, and missing international classification of diseases-9 (ICD-9) code at discharge in the electronic records.

### 2.3. Framework Development

#### LLM-Based Framework Development

**Figure 1:**
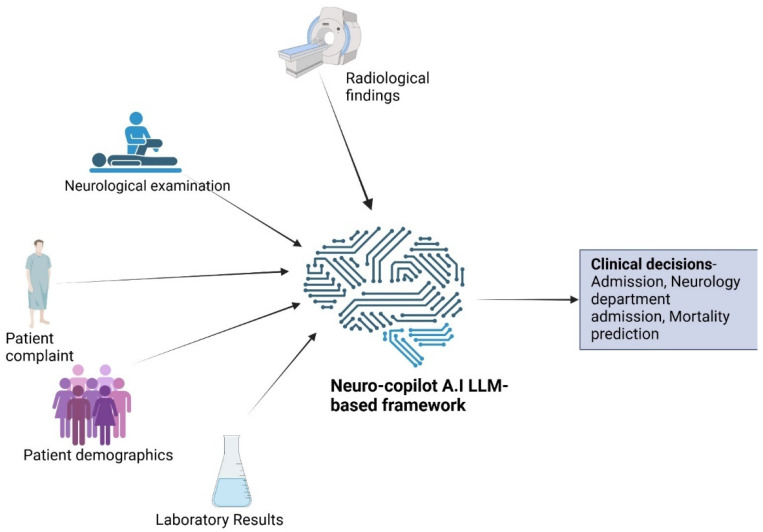
Inputs and outputs of Neuro AI large language model (LLM)-based framework—The model accepts patient anamnesis (medical history), findings from the neurological examination, patient demographic (age and gender), radiological findings (as free text), and laboratory findings (as tabular data).

For the LLM component, we leveraged the Gemini 1.5-pro LLM through a securely hosted API on Google Cloud’s Vertex AI platform, with strict data management agreements ensuring that patient information is used solely for research purposes and never for retraining the model. The LLM was configured with default parameters (temperature = 1, top_*p* = 1, *n* = 1). The model performed two principal functions: (1) providing numeric scores (1–7 for general likelihood of a positive response and 1–100 for mortality prediction, where 100 indicates a 100% likelihood of death within a specified timeframe) and (2) generating English-translated summaries with recommended next steps. The inputs included both structured and unstructured data extracted from electronic health records (EHRs)—encompassing neurological examinations, patient medical history, radiological findings, and laboratory results (Figure 1).

To improve performance, we employed prompt engineering strategies (priming, chain of thought, and self-consistency) and integrated a retrieval-augmented generation (RAG) module using the all-miniLM-L6-v2 model to create dense vector embeddings. The Faiss library retrieved the five most relevant historical cases from the dataset based on cosine similarity, ensuring no bias by excluding the specific patient case under comparison (i.e., the one with a similarity score of 1). This RAG data spanned demographics, neurological findings, ICD-9-coded conditions, and hospitalization outcomes. Additionally, to bolster reliability, the LLM was run five times per query, with the final numeric score calculated as the mean of these runs and the first generated text response retained for subsequent analysis. For comparison of the LLM compartment, we evaluated the framework compared to an LLM baseline without prompt engineering, RAG, or increased query frequency. The complete prompts are provided in the Supplementary Materials.

### 2.4. Machine Learning Framework Development

We integrated machine learning techniques with our LLM-based approach. To harness the unstructured data processed by the LLM, we utilized LLM-generated translated patient information as inputs for a logistic regression classifier. We employed TF-IDF vectorization to handle the unstructured free-text format effectively, while an XGBoost model was incorporated to manage the tabular data. XGBoost was specifically used due to the complexity of missing values arising from the diversity of laboratory tests. Both logistic regression and XGBoost hyperparameters were tuned using 5-fold cross-validation, optimizing for AUC through grid search. The optimal configurations were eXtreme Gradient Boosting (XGBoost) with a learning rate of 0.1, max depth 3, and 50 estimators, and logistic regression with L2 regularization (C = 1.0) and “liblinear” solver.

The machine learning framework (0–1 prediction) was built to predict together with LLM numeric prediction’s part (1–7 score).

Data processing and feature engineering: categorical variables (ECG/CT/MRI/Sex) were encoded with a stable integer map (unknowns = −1). Numeric laboratory values were kept continuous; no imputation was applied for the tree-based model (XGBoost natively handles missing values). English-language summaries were lower-cased; punctuation and digits removed; tokens generated with natural language toolkit (NLTK); English stop-words removed; WordNet lemmatization applied; and features constructed with term frequency–inverse document frequency (TF-IDF) (max_features = 10,000). Random seeds were fixed for all model training and evaluation.

### 2.5. Ensemble Framework Development

In this ensemble approach, we combine the LLM’s numeric output (scored 1–7) with the machine learning models’ binary (0–1) predictions. To facilitate this integration, we first converted the LLM outputs into binary classification probabilities by applying min–max normalization, scaling the scores to a 0–1 range. We then implemented a standard train–test split, allocating 80% of the dataset for model training and 20% as a fixed holdout for performance evaluation. The machine learning models, XGBoost and Logistic Regression, were trained on the 1118 less-detailed cases (i.e., the “training” set). The performance was evaluated on the 250 comprehensive cases (i.e., the “test” set). Importantly, the LLM-based framework required no training or fine tuning and, therefore, was tested only without training on the test dataset

**Figure 2:**
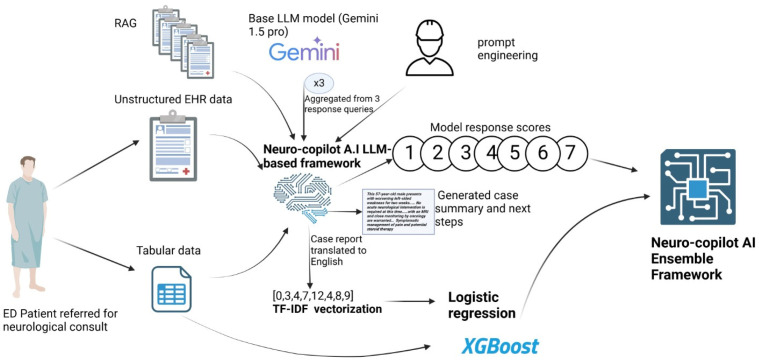
Schematic representation of the Neuro ensemble framework. The system employs a large language model-based (LLM-based) approach to integrate unstructured electronic health record (EHR) text and structured tabular inputs through a combination of retrieval-augmented generation (RAG), feature vectorization (e.g., term frequency–inverse document frequency (TF-IDF)), and multiple modeling methods—including logistic regression and eXtreme Gradient Boosting. These outputs are aggregated and refined to inform the next steps in patient management, thereby enhancing the accuracy and clinical utility of neurological consultations in the emergency setting.

To generate the final ensemble output, we combined the probability estimates from all three models. Model-specific weights that sum to 1 (e.g., an equal weight of 0.33 for each model). We optimized weights w_1_, w_2_, and w_3_ on a validation split by grid-searching the probability simplex in 0.1 increments to maximize receiver operating characteristic curve (AUC) and then evaluated the fixed weighted average on the held-out test set. This weighted combination ensures a robust and balanced classification outcome by leveraging the strengths of each individual model (Figure 2). To assess the added value of this approach, we compared the ensemble framework against the standalone LLM framework and the traditional machine learning frameworks to determine whether ensemble integration improved overall performance.

## 3. Overview of Analyses

The primary analysis focused on the 250 comprehensive cases (18.27%), ensuring sufficiently detailed clinical data for evaluating the LLM-based (and consequently ensemble) performance. The remaining 1118 cases (81.73%) were used to train the machine learning models. A random subset of 100 cases, drawn from the test dataset, was independently reviewed by three senior neurologists, enabling a direct comparison of AI-driven predictions with expert clinical judgment and further illustrating the potential benefits of the ensemble framework.

### Statistical Analysis

For comparisons between groups, qualitative variables were analyzed using Fisher’s exact test and chi-square test. Continuous variables that followed a parametric distribution were analyzed by Student’s *t*-test, and nonparametric variables were analyzed by the Mann–Whitney U test. Agreement between model responses was measured using a Fleiss’ Kappa coefficient for categorical agreement and an Intraclass Correlation Coefficient (ICC) two-way random-effects model for absolute agreement (ICC2, k) to assess the reliability of the three raters. This method was also used to assess the accuracy of subjective labels such as admission, with three senior neurology physicians serving as raters. Pearson’s correlation coefficient was used to evaluate the strength and direction of the relationship between the model’s predictions and the experts’ average scores. We evaluated predictive model efficacy, leveraging the area under the curve- receiver operating characteristic (AUC-ROC). For the AUC-ROC, we report 95% confidence intervals (CIs) computed via stratified bootstrap (2000 resamples) that preserves the empirical class balance in each resample. The threshold for significance was set at *p* < 0.05.

## 4. Results

### 4.1. Cohort Characteristics

To evaluate Neuro AI’s capabilities in making clinical decisions, we identified 1368 consecutive cases of patients who underwent neurological consults at the ED (Table 1). All cases had neurological assessment per inclusion criteria with additional testing, including brain computed tomography (CT, *n* = 1075, 78%) or magnetic resonance imaging (MRI, *n* = 43, 3%), blood tests (*n* = 1261, 92.17%), urine tests (*n* = 215, 15.71%), and cerebrospinal fluid (CSF) analysis (*n* = 69, 5.04%). Out of the cohort, 625 patients were admitted (45.7%) and 428 (31.35%) of them admitted to the neurological department. Seven hundred twenty-eight patients (53.2%) were discharged from the ED with an ICD-9 code indicating diseases of the nervous system, while 640 patients (46.8%) were discharged with a diagnostic code unrelated to neurology. To assess the skewness or bias of the data, statistical analysis was performed. The only bias noted was that higher night shift consultations were observed among patients admitted (36% vs. 18.7%, *p* < 0.001). This suggests a tendency to admit more during the night shift. The remaining variables, such as higher mortality (13.7% vs. 2.69%, *p* < 0.001), older age (68.6 IQR vs. 48, *p* < 0.001), shorter time to neurological consultations (1.91 ± 2.51 vs. 2.27 ± 2.21 days, *p* < 0.001), and a higher acute stroke diagnosis (44% vs. 3.49%, *p* < 0.001), were statistically more common in the admitted group. These findings suggest that the cohort aligns with the known characteristics of admitted patients, indicating no additional bias in the cohort.

### 4.2. Admission Prediction

**Figure 3:**
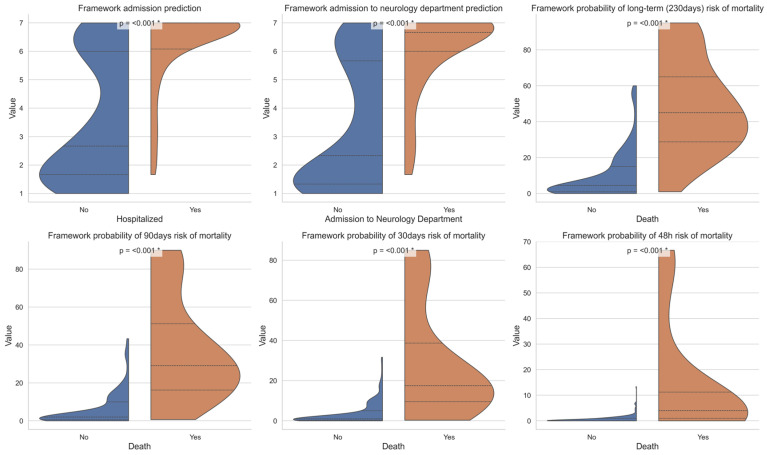
LLM scores for admission and mortality—Violin plots show the distribution of the LLM output scores for admissions and mortality, stratified by the actual outcome. The blue (No) and orange (Yes) are real-life decision outcomes matched with the scale 1–7 of the model ranking. *—Statistical significance (*p* < 0.005).

**Figure 4:**
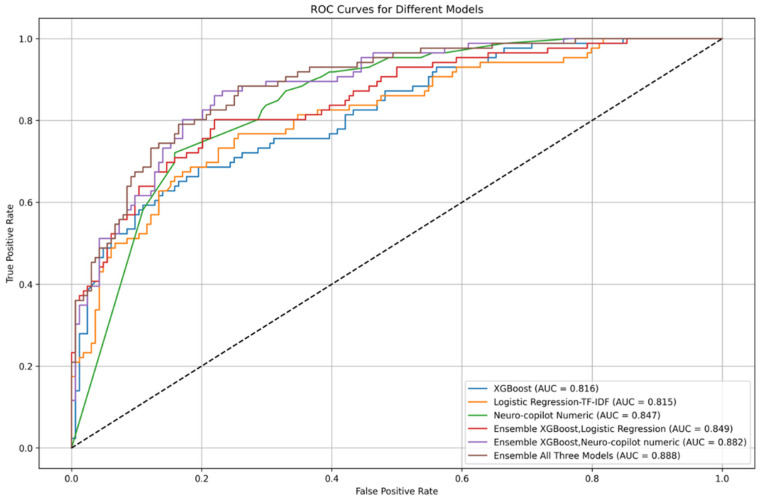
Receiver operating characteristic (ROC) curves for individual and ensemble models: XGBoost (AUC = 0.805), logistic regression-TF-IDF (AUC = 0.846), Neuro-Copilot Numeric (AUC = 0.824), and ensemble of all models (AUC = 0.871). Neuro-Copilot Numeric is the rating of the LLM-based framework from 1 to 7. All ROC curves were analyzed in the test set.

The LLM and ML frameworks were tasked with predicting admission and specifically admission to the neurological department based on findings that were collected at the ED; the AI was blinded to the outcome itself. For the 250-patient analysis, the base LLM achieved an area under the curve receiver operating characteristic (AUC-ROC) of 0.79 for admission prediction across the entire cohort. Upon using the Neuro-Copilot AI LLM-based framework, the AUC-ROC improved to 0.847, with a Fleiss’ kappa of 0.52. For cases where the real-world decision was discharge, the admission scores showed a bimodal distribution, with a smaller peak observed at score 6 (Figure 3, indicating the tendency of LLMs to have a slight bias towards admission. To further enhance our framework, we integrated an LLM-based framework with traditional ML. First, we transformed the English-translated case reports into a numeric vector through TF-IDF and trained a predictive model of admission on the training set using logistic regression. Using the probabilities, this approach improved AUC-ROC very slightly to 0.849 [95% CI 0.795–0.894]. Next, we applied an ensemble approach that combined the LLM, logistic regression, and XGBoost, which further improved the prediction to 0.888 [95% CI 0.846–0.926] on the test cohort (Figure 4 and Supplementary Material S1). The optimal weights were XGBoost: 0.40; logistic regression: 0.20; and admission score: 0.40.

To provide explainability besides the numeric responses and probabilities, the Neuro-Copilot AI LLM-based framework was tasked with providing both a summary of the case and the next best steps. These summaries were transformed through TF-IDF and analyzed using a logistic regression model. TF-IDF analysis identified terms like “stroke”, “therapy” (implying tissue plasminogen therapy, tPA), and “immediate” linked with admission (Supplementary Table S1). This finding emphasizes the high prevalence of stroke cases and their significant association with admission status. We further evaluated whether our framework can specifically predict admissions to the neurology department. The framework achieved an AUC-ROC of 0.837 for the test cohort, with a Fleiss kappa of 0.45. Using the ensemble approach, the AUC-ROC improved to 0.868 [95% CI 0.817–0.890] (Supplementary Figure S1 and Supplementary Material S1). The optimal weights were XGBoost: 0.10; logistic regression: 0.40; and admission score: 0.50.

### 4.3. Mortality Prediction

**Figure 5:**
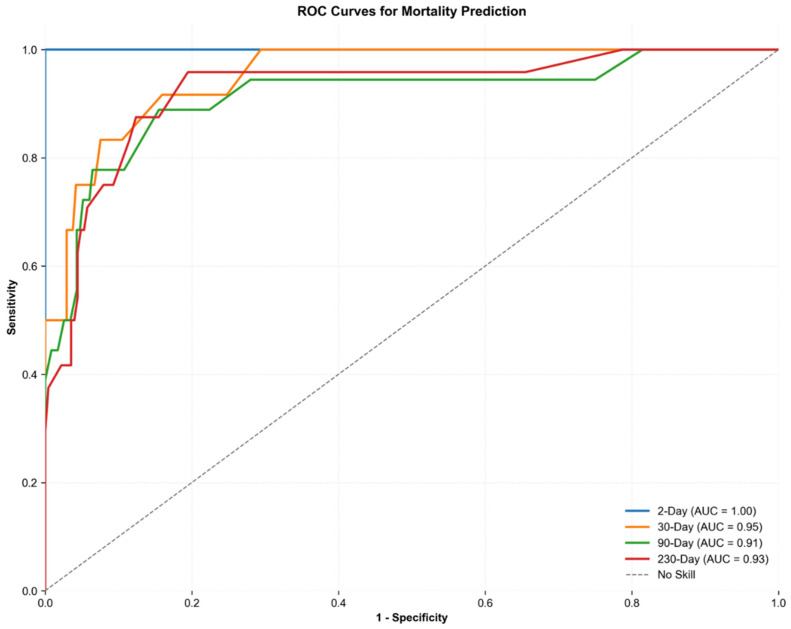
Time-dependent ROC curve and PRC curve for mortality displays precision—recall and ROC curves for 2-day, 30-day, 90-day, and 230-day mortality predictions.

To evaluate the ability to predict patients at high risk for mortality, the model was tasked with estimating the probability of mortality at key time points: 48 h, 30 days, 90 days, and the final follow-up at 230 days. In total, up to the last day of follow-up, 24 patients died (9.6%). A time-dependent AUC-ROC curve assessed the framework performance. For the 250-patient analysis, the base LLM demonstrated strong performance in the mortality risk prediction, achieving an AUC-ROC of 0.93 for long-term mortality (230 days) and 0.99 for short-term mortality (2 days). Notably, the Neuro-Copilot AI LLM-based framework consistently outperformed the base LLM across all time points (ranging from 0.91 to 1, Figure 4)**.** Due to the low number of deaths, we did not attempt to use our ensemble approach for this task as it would be too low for assessment on the test set. The LLM-based framework consistently predicted lower mortality probabilities, particularly with respect to short-term mortality risk (Figure 5).

### 4.4. Comparison to Expert Assessments

To assess agreement between the LLM-based framework, expert decisions, and clinical outcomes, three human senior neurologists’ (GT, RW, and SP) manually reviewed 100 randomized cases from the 250 cases, blinded to the actual decision and the LLM outputs. We converted the LLM output scale (1–7) and expert opinions to binary values (admit or not) using a threshold of 4. The average prediction AUC-ROC of the experts with the actual decision was 0.87. In comparison, the framework achieved an AUC-ROC of 0.91. We noted that the Fleiss’ kappa for admission predictions by the experts was only 0.21, indicating slight agreement, reflecting the inherent subjectivity in clinical admission decisions. However, Neuro-Copilot AI predictions were correlated with the average expert score, with a Pearson correlation coefficient of 0.79 (*p*-value < 0.001), suggesting that while expert opinions were varied, the model consistently aligned with their collective judgment. Notably, in the 18 cases where all three neurologists were in full agreement on admission likelihood, Neuro-Copilot AI predictions matched the experts in all cases. For mortality predictions, both Neuro-Copilot AI and the neurologists were tasked with providing a probability of mortality over the next 230 days. The average AUC-ROC of the experts was 0.91, in comparison to Neuro-Copilot AI, which achieved an AUC-ROC of 0.90. Interestingly, The ICC for mortality prediction among the experts was 0.81, demonstrating great consistency and reliability. A possible explanation for the similar AUC-ROC results is the fact that future mortality is easily identified in the patient cases presented to the experts.

## 5. Discussion

We introduce a robust AI-driven framework designed to assist neurologists in the ED. It excels in critical predictions, including admission and mortality risk. The framework achieved an AUC of 0.84 for admission prediction, improving to 0.88 for general admission and 0.86 for neurological department admission when combined with traditional machine learning algorithms. Such high AUC is impressive given the notion that the AI models were blinded entirely to the physicians’ decisions regarding admission status and mortality status of the patient. Notably, the framework combines structured data with LLM-generated inference outputs, resulting in enhanced predictive accuracy.

Currently, none of the existing studies specifically address neurology-focused prediction models, but prior work has explored generalized models for ED settings. For instance, the NYUTron model developed by Jiang et al. achieved an AUC of 0.94 for in-hospital mortality prediction [22]. Similarly, Glicksberg et al. demonstrated an AUC of 0.87 for admission predictions using Bio-Clinical-BERT alongside XGBoost, which is surpassed by the Neuro-Copilot AI ensemble approach [23]. Generative models excel in predicting mortality, probably due to their ability to capture the complex interplay of comorbid conditions indicative of poor clinical outcome, as well as the nature of mortality as a ground truth. In contrast, admission predictions, while accurate, show greater variability (Fleiss’s kappa = 0.59 for admission compared to ICC = 0.97 for mortality) This variability reflects the subjective and context-dependent criteria for admission, which lack a universal standard. Improving admission prediction requires refining how admission labels are defined. For example, patients discharged but returning for admission within three months may signal an initial discharge error. Likewise, a patient’s death within three months could indicate a missed opportunity for earlier intervention. Short hospital stays of 1–2 days may reflect physician uncertainty in discharge decisions.

In our study, not surprisingly, hospitalized patients tend to be older; have higher priority levels; and present with more severe neurological conditions, particularly stroke. Their care involves more frequent imaging; laboratory tests; and specialized treatments, such as tPA and antiplatelet therapy. Mortality is significantly higher in this group. In contrast, discharged patients are more likely to have headaches or neuromuscular complaints bringing them to the ER, received fewer diagnostic tests, and are more frequently treated with symptomatic medications like opiates. It is known that human judgment is inherently prone to cognitive biases and limitations, making tools that augment decision-making, such as AI, valuable in improving outcomes and reducing errors. Integrating data systems designed to support neurologists in the ED is not only valuable but inevitable. Such systems have the potential to leverage unstructured EHR data more effectively, improving both clinical decision-making and operational efficiency.

Our study has important limitations. It was conducted at a single center over a short, two-month period and used a retrospective design, which limits generalizability. The ensemble framework was evaluated on a small test set (*n* = 250); optimizing model weights on such a sample increases the risk of overfitting and instability to case-mix. The diagnostic distribution in the test cohort, particularly the high prevalence of stroke, a condition strongly associated with admission, may have amplified apparent performance and reduces validity for less frequent neurological presentations. We did not perform external validation on an independent or multicenter cohort. In addition, the outcome used as a “gold standard” (hospital admission) is inherently subjective and context-dependent, influenced by organizational factors (e.g., staffing and bed availability) and clinician preference. There are also minor concerns. Automated extraction from electronic records may omit relevant contextual variables (social determinants, organizational pressures, and attending experience), leading to incomplete clinical data. Model explainability remains partial: although we provide textual rationales and TF-IDF term importance, it is unclear whether these are consistently interpretable and clinically useful in contentious scenarios. Finally, beyond the observed night-shift effect, other potential systematic biases (e.g., by sex or socioeconomic status) were not examined.

Our study directly enhances ED neurology efficiency by streamlining decision-making. This framework can reduce patient length of stay, a key challenge in neurological consultations for maintaining or improving care quality. This is a necessary step toward optimizing efficiency in neurology primary care [24]. Additionally, in hospitals with limited access to neurologists, our framework could theoretically serve as a valuable support. A study by Kachman et al. identified key areas where AI could enhance clinical operations in the ED^16^, and Neuro-Copilot AI specifically addresses three critical functions: reviewing EHR data, assisting with documentation, and augmenting clinical decision-making. However, the most difficult to implement is decision augmentation, as neurologists typically have already formed their conclusions by the time they consult decision-support tools. This conflict can be exacerbated by cognitive biases, such as anchoring bias^6^, where clinicians may overly rely on their initial impressions, making them less receptive to alternative suggestions. The ensemble framework is designed to provide transparent insights and reasoning, ensuring each decision is accompanied by clear explanations.

Future research should prioritize refining these models within real-world clinical settings and assessing the combined accuracy of AI models and medical professionals working together rather than assessing AI performance in isolation. Collaborative methodologies, such as clinical trials and prospective studies in ED settings, will be essential for their successful integration into clinical practice.

## Figures and Tables

**Table 1:** Patient demographics and hospitalization outcomes.

Features	Total (*n* = 1368)	Hospitalized (*n* = 625)	Discharged (*n* = 743)	*p*-Value
Age at arrival to emergency department (ED) (median, interquartile range (IQR))	58.6 [38.37–74.5]	68.6 [51.9–77.7]	48 [31.5–67.5]	**<0.001**
Male (*n*, %)	663 (48.46%)	335 (53.6%)	328 (44.14%)	**<0.001**
Mortality	106 (7.74%)	86 (13.7%)	20 (2.69%)	**<0.001**
Time from arrival to ED to mortality (Days) (M ± standard deviation (SD))	56.91 ± 57.35	52.90 ± 58.03	74.15 ± 52.20	**0.028**
Time from arrival to ED to neurological consult (Hours) [M ± SD]	2.1 ± 2.4	1.91 ± 2.59	2.27 ± 2.21	**<0.001**
Number of neurological consults after 19:00 until 8:00 (Night shift)	364 (26.6%)	225 (36%)	139 (18.7%)	**<0.001**
Priority level 1 (P1)	142 (10.38%)	121 (19.36%)	21 (2.82%)	**<0.001**
ICD-9 code with diseases of the nervous system at release from ED	728 (53.21%)	416 (66.56%)	312 (41.99%)	**<0.001**
Neurological category of consult based on ICD-9 code on release				
Stroke and cerebrovascular disorders	301 (22%)	275 (44%)	26 (3.49%)	**<0.001**
Seizure disorders	141 (10.3%)	62 (9.92%)	80 (10.76%)	0.67
Headache disorders	179 (13%)	18 (0.16%)	161 (21.66%)	**<0.001**
Neuromuscular disorders	45 (3.28%)	11 (1.76%)	34 (4.57%)	**<0.001**
Central demyelinating disorders	16 (1.16%)	15 (2.4%)	1 (0.13%)	**<0.001**
Infections of the nervous system disorders	13 (0.95%)	12 (1.92%)	1 (0.13%)	**0.001**
Neurodegenerative disorders	5 (0.36%)	3 (0.48%)	2 (0.26%)	0.84
Other disorder of central nervous system (CNS)	28 (2.04%)	21 (3.36%)	7 (0.94%)	**<0.001**
International Classification of Diseases-9 (ICD-9) code without diseases of the nervous system and sense organs at release from ED	640 (46.78%)	209 (33.44%)	431 (58%)	**<0.001**
Imaging conducted at ED				
Computed tomography (CT)	1075 (78.58%)	558 (89.28%)	517 (69.31%)	**<0.001**
Magnetic resonance imaging (MRI)	43 (3.14%)	43 (6.88%)	0	**<0.001**
Electrocardiography (ECG)	836 (61.1%)	515 (82.4%)	321 (43.2%)	**<0.001**
Laboratory data				
Blood test	1261 (92.1%)	617 (98.7%)	644 (86.6%)	**<0.001**
Lumbar puncture	69 (5.04%)	45 (7.2%)	24 (3.23%)	**0.001**
Urine test	215 (15.71%)	126 (20.16%)	89 (11.97%)	**<0.001**
Treatment data				
Tissue-type plasminogen activator	29 (2.11%)	29 (4.64%)	0	**<0.001**
Opiate drugs	39 (2.85%)	8 (1.28%)	31 (4.17%)	**0.002**
Triptan drugs	1 (0.07%)	1 (0.16%)	0	**1**
Corticosteroid therapy	71 (5.19%)	47 (3.43%)	24 (3.23%)	**<0.001**
Anticonvulsant drugs	151 (11.03%)	92 (14.72%)	59 (7.94%)	**<0.001**
Antibiotic drugs	68 (4.97%)	58 (9.28%)	10 (1.34%)	**<0.001**
Antiplatelet drugs	144 (10.52%)	135 (21.6%)	9 (1.21%)	**<0.001**
Antiemetic drugs	166 (12.13%)	65 (10.4%)	101 (13.59%)	0.085
IV fluid therapy	351 (25.65%)	168 (26.88%)	184 (24.76%)	0.37

## Data Availability

The data underlying this article will be shared on reasonable request to the corresponding author.

## Supplementary Materials

The following supporting information can be downloaded at: https://www.mdpi.com/article/10.3390/jcm14176333/s1, Figure S1: ROC Curves for individual and ensemble models in predicting neurological department admissions; Table S1: Odds ratios (OR) and mutual information (MI) values for words linked to admission, sorted by highest mutual information values; Material S1: Optimized Weights for admission task. Material S2: Neuro-Copilot LLM based-framework Prompt.
